# P-1167. Pediatric Coccidioidal Meningitis: a 39-year Single-Center Retrospective Cohort

**DOI:** 10.1093/ofid/ofae631.1353

**Published:** 2025-01-29

**Authors:** Lienna Y Chan, Jordan Gotwalt, Marian Lacy, Nathan B Price, Scott C Olson, Neil M Ampel, Kareem W Shehab

**Affiliations:** University of Arizona, Tucson, Arizona; University of Arizona College of Medicine - Tucson, Tucson, Arizona; University of Arizona College of Medicine, Tucson, Arizona; University of Arizona, Tucson, Arizona; University of Arizona, Tucson, Arizona; University of Arizona, Tucson, Arizona; University of Arizona, Tucson, Arizona

## Abstract

**Background:**

Coccidioidal meningitis (CM) is a rare complication of coccidioidomycosis, an endemic mycosis of the southwestern United States. CM is associated with significant morbidity and mortality, but its clinical characteristics and outcomes are poorly described in children. Herein we present a 39-year retrospective cohort of pediatric CM at a single academic center in the endemic area.

**Methods:**

We conducted retrospective chart review with IRB approval of all patients from birth to 18 years of age with CM from 1981-2010 and 2013-2023. Demographic and clinical data, including presenting signs/symptoms, initial lab and imaging results, treatment course, and complications were collected and summarized.

**Results:**

Sixteen patients with CM were identified. The most common presenting symptoms included headache (50%), fever (44%), nausea/vomiting (38%), weight loss (31%), and cough (19%). Ten patients (63%) initially presented with CM, while 6 patients (38%) presented with CM between 2 weeks to 4 years after initial diagnosis of primary pulmonary coccidioidomycosis. Of 14 patients with serologies, 100% had a positive IgG and 43.8% had positive IgM. Of 14 with an initial CSF sample, 12 (86%) had pleocytosis (median 114.5, range 7-690 WBC/mm^3^). Of 13 with initial complete blood counts, four (30.7%) had leukocytosis and one had eosinophilia. Hydrocephalus requiring ventriculoperitoneal shunt (VPS) developed in 13 patients (81.3%); two died before a VPS could be placed and one developed CM without hydrocephalus 4 years after initial diagnosis. Of 15 patients prescribed lifelong therapy, 14 received fluconazole and one received voriconazole. Two received IV amphotericin B during their course.

**Conclusion:**

Most children with CM presented without recognized primary pulmonary coccidioidomycosis, with 50% presenting with headache and fewer than 50% with fever. Most patients had CSF pleocytosis without peripheral leukocytosis. Nearly all were treated with fluconazole. Death occurred in two patients; all survivors required VPS. Future prospective trials examining higher doses of fluconazole and use of mold-active azoles are needed to assess their efficacy in preventing hydrocephalus in children with CM.

**Disclosures:**

**Nathan B. Price, MD**, DSM-Firmenrich: Advisor/Consultant

